# Congenital Malformations

**Published:** 1904-04-02

**Authors:** 


### Congenital Malformations.

Abnormalities which date from intra-uterine life are of perennial interest, and hence it is not a little curious that so few theories of causation have been propounded. Nine out of ten text-books of pathology, when dealing with any particular region of the body, commence their account with a description of the congenital abnormalities that may occur in that part. But they usually omit any discussion of the etiology, beyond a passing reference to heredity as a causal factor. The theory of maternal impressions is not, generally speaking, treated seriously by the profession, although recently there have appeared in the medical journals some curious reports bearing on the question. Thus there was the Jewess, who during pregnancy brooded very much over the expense that would be incurred of having the baby circumcised should it turn out to be a boy, and who mentioned her worry to the medical attendant before the birth of the child. The baby proved to be a boy, but, oddly enough, there was no foreskin. Again we hear of a lady being frightened by a tortoise, and subsequently bearing a child with webbed fingers. Such cases are hardly likely to be regarded as anything more than interesting coincidences. More consideration is to be given to hereditary influences. Indeed, there is little doubt that malformed parents are more likely than normal ones to beget children having some or other physical defect, and true as this is as a general proposition, it is also certain that the particular deformity from which the parent suffers is especially liable to appear in his children; a fact which is shown with comparative frequency in hare-lip and cleft-palate. It may, nevertheless, be somewhat rash to speak of heredity as the essential cause. It is no doubt a factor, but the actual determining agent may be something quite independent of this; some external influence, perhaps, acting upon the mother before or during pregnancy, which yet requires a certain predisposition in order that a malformation may appear in the offspring. A remarkable case was recently shown by Dr. Cautley before the Society for the Study of Disease in Children. The patient was a child in whom there were present at least seven distinct congenital malformations. In the right eye there was a subconjunctival dermoid, while there was a cleft in the upper lid of the left eye and a coloboma of the iris, which also involved the choroid. There was right-sided macrostoma, accessory auricular appendages on each side, one, if not both, containing cartilage. A sacro-coccygeal dimple was present, and the child was unable to stand or walk. If heredity be considered as the main cause instead of merely a predisposing factor it would have been more than justifiable to expect in this instance a family history of similar tendencies towards imperfect development. This, however, was not so. The patient was the eleventh child of a family of twelve, and in the other eleven, of whom nine were living, no congenital deficiency had been observed; nor could a history be obtained of such occurring among the parents or their relatives. It seems, therefore, that some other factor than heredity was mainly responsible, and it would be of the utmost interest and advantage to know what such factor might be. That the problem may not be altogether beyond the reach of practical inquiry seems indicated by the observation recently made by Dr. Hewetson of an apparent connection between congenital goitre and the administration of potassium chlorate to the mother during pregnancy.

We believe that there is a great need of more vigorous research into the whole question of external influences affecting fœtal development, including as it does not only the production of malformation but also the determination of sex. It is certain that a fuller knowledge of such matters would entail great benefits to mankind.

